# Research on the Prediction of Popularity of News Dissemination Public Opinion Based on Data Mining

**DOI:** 10.1155/2022/6512602

**Published:** 2022-03-29

**Authors:** Jing Tian, Huayin Fan, Zengwen Hou

**Affiliations:** Journalism & Communications College, Jilin Normal University, Changchun 130012, Jilin, China

## Abstract

The development of news public opinion presents the characteristics of dynamic changes, and its life cycle is generally relatively short. For news public opinion to be welcomed by everyone, that is, to become hot news, it must be able to spread to a large number of readers in a short time. And, some of its characteristic attributes must satisfy the interests of most users and arouse users' desire to read. Therefore, it is particularly important to extract and study these characteristic attributes that determine the popularity of news public opinion and finally establish a model to describe the relationship between the popularity of news public opinion and these characteristic attributes. Based on data mining, this article mainly studies the popularity of news public opinion from two aspects. First is the sampling and attribute feature extraction of news. Then, considering the nonlinear relationship between the features, an improved principal component analysis method is proposed to analyze the correlation of the features. This can select important features from many irrelevant features and effectively reduce the original high-dimensional features. Second, the application of neural network in the prediction of news public opinion is studied. BP is an efficient data mining method. Considering BP network has some shortcomings. This work uses an improved particle swarm optimization to optimize the initial parameters for BP network, which can compensate for the defects of BP network. After that, the BP network with optimized parameters is used to establish a prediction model for the popularity of news public opinion. The experimental results prove that the neural network model proposed can accurately predict the popularity of news public opinion.

## 1. Introduction

The term news was first born in the Tang Dynasty and refers to the time that was recently heard or what happened recently in society. The meaning of the word news has not changed much after more than a thousand years of development. However, the dissemination of news has undergone earth-shaking changes after more than a thousand years of historical changes. Traditional news dissemination, from small-scale oral transmission to newspapers, to wireless broadcasting, and to TV broadcasting, blooms in many ways and has various channels. Nowadays, the news is presented on everyone's mobile terminal, and traditional news dissemination methods have gradually withdrawn from the market [1–5].

With the rapid development of the Internet, the Internet has become a part of people's lives. People use mobile phones to receive information and view news anytime and anywhere on the Internet. Compared with traditional news dissemination, the Internet not only brings more traffic but also shortens the generation, creation, and dissemination time of news exponentially. The data shows that more than 2,600,000 news are shared every minute on Facebook, the average number of online videos shared every minute on LINE is 8,333, and the number of news links shared every minute on Twitter is close to 300,000. Interconnection has detonated the information age, and news hotspots have been generated all the time. Traditional news dissemination is restricted by channels, and the audience is limited. Even if it is a hot topic, its influence is also limited. But nowadays, the coverage of breaking news is so wide that it is unimaginable [6–10].

In the Internet age, news and public opinions cover all aspects of our lives. The real-time, uncertain, and rapid spread of the Internet make the characteristics of online news public opinion show the characteristics of the new era such as randomness, suddenness, and concealment. In such an era, everyone on the Internet can become a publisher and disseminator of information, as well as a creator of news and public opinion. Of course, news and public opinion are not all good, and the domestic Internet environment is not a pure land, which is full of negative, distorted, extreme, inciting violence and division factors. Popular news, especially breaking news, as a powerful means of communication, is not only conducive to the rapid dissemination of information but also conducive to the transfer of value. Under such a background, predictive analysis of the popularity of news and public opinion is beneficial to the healthy development of the Internet and curbs negative information such as rumors and violence, at the same time, producing news or advertisements that can arouse people's general goodwill. It has great value for many Internet publishers, portal websites, and Internet advertising companies. The development of news public opinion presents the characteristics of dynamic changes, and its life cycle is generally relatively short. If a piece of news is to be welcomed by everyone, it becomes hot news. It must be able to spread to a large number of readers in a relatively short period of time, and some of its characteristic attributes must satisfy the interests of most users and arouse users' desire to read. Therefore, it is particularly important to extract and study these characteristic attributes that determine the popularity of online news, analyze their interrelationships, and finally, establish a model to describe the relationship between the popularity of news public opinion and these characteristic attributes [11–15].

The contributions are as follows: (1) This work proposes an improved principal component analysis method to analyze the correlation of features. In this way, important features can be selected from many irrelevant features, effectively reducing the dimensionality of the original high-dimensional features. (2) This work uses an improved particle swarm optimization to optimize the initial parameters for the BP network. It can make up for the shortcomings of BP and use the BP network with optimized parameters to establish a prediction model for the popularity of news and public opinion.

## 2. Related Work

Compared with traditional news and advertising industries, Internet news, with its low-cost, fast-spreading, and wide-ranging features, had a huge impact on the traditional news industry and had shown initial advantages. Due to the real-time characteristics of news and the guiding characteristics of public opinion, accurately predicting the popularity of a news manuscript for the roles of journalists, content providers, advertisers, etc., had become a new research hotspot. However, predicting the popularity of news articles on the Internet was a challenging task. First of all, the content of these articles was only available online. The structure of the social network that spreads news was different, which would affect the probability that users can see this article. In addition, the coverage of the content of the article was also a factor that affects the prediction of the results. For example, articles that were closely related to the general public on a global scale are more popular than articles that only targeted a small number of social groups.

For this challenging work, a lot of research had been devoted to it, and a lot of questions had been raised. Literature [16] put forward the problem of tracking the spread of online topics. Literature [17] proposed detecting the public opinion orientation of online communities. The literature [18] put forward the field of prediction of the popularity of social media. There were many different ways of expressing the concept of popularity. For example, our most common classic method was the number of clicks on a piece of news, but this information was of little significance to researchers. Because in fact, due to the existence of web crawlers and search engines, it was difficult for us to estimate the number of times, an article was actually requested by users, which would directly lead to the inaccuracy of the original data. In addition, there were some other methods based on the user's own participation, such as comments, voting, and sharing via email or WeChat. Predicting the popularity of news articles was a difficult task, and a lot of research results had been accumulated over the years. Literature [19] proposed a model to predict the popularity of news published by science and technology information websites. This method believed that the popularity of articles will gradually increase over time. Literature [20] proposed two algorithms for predicting video popularity and news popularity and achieved good prediction results. Literature [21] proposed a linear logarithm method, which can meet the forecasting demand in a specific range. Literature [22] proposed a different way of thinking. He established a new prediction model based on the social influence of news and the characteristics of the publishing platform. The disadvantage of this type of algorithm was that it could only be applied to predictive analysis in a specific field but failed to analyze articles on different topics. Literature [23] proposed a new forecasting idea, which focused on the prediction of the popularity of certain news that would continue to be paid attention to after a certain period of time. Literature [24] proposed a method. Using the number of news sharing as a popular indicator, a clustering algorithm was used to divide different articles into 4 categories according to their popularity. Literature [25] proposed a new idea to predict the popularity of online news and ranked it according to the popularity. The disadvantage of this type of method was that it could not give an accurate prediction value.

Driven by the Internet industry chain, Internet + had become an industrialized concept, and network news was quickly spread through the Internet as a carrier. Data showed that more than 26,000 news were shared per minute on Facebook, the average number of online videos shared per minute on LINE was 8333, and the number of news links shared per minute on Twitter was close to 3,000. Clarifying how popular the public was for a piece of news has become a new branch of research.

At present, many models for tracking and forecasting online news based on time series had been proposed. These models assumed that the evaluation of popularity was time-sensitive. The number of users of YouTube videos fluctuated continuously within 24 hours, and the number of their shares was also constantly changing. The literature [26] aimed at analyzing the life cycle of online news; this research used a threshold value to compare and calculate the number of news shares in 7 days. At the same time, the feature extraction method was another subtle branch in the field of social news research. It mainly focused on the news itself and removed some possible influence factors, such as time, comment, and emotional color. The reason why time was eliminated was that the number of news sharing gradually increases with the passage of time. But its impact on users would gradually become zero after a certain point in time. In addition, the results of this research field were diversified because of the existence of various media forms, including videos, music records, and plain text information [27].

## 3. Method

### 3.1. Improved Principal Component Analysis

#### 3.1.1. Algorithm Background

When predicting the popularity of news public opinion, it is necessary to extract the characteristics of the news. Under normal circumstances, these features are high-dimensional, and processing these features is more complicated. Therefore, it is necessary to reduce the dimensionality of the features before making subsequent predictions. Principal component analysis has become a classic and effective statistical dimensionality reduction method. It has been successfully applied to linearly distributed data for a variety of practical problems and has also achieved remarkable results. At the same time, the algorithm for further expansion of principal component analysis has also been effectively developed.

First of all, through research, we can know that sparse principal component analysis is based on principal component analysis, which mainly transforms the problem of solving the principal component load into a ridge regression problem. A regularization penalty term is added to the essence of such an optimization regression problem to compare the load of each principal component. This promotes a load of some relatively weak variables to approach zero, achieving a sparse effect. At present, there are generally two types of penalty items in sparse principal component analysis: *L*_1_ penalty items and *TL*_1_ penalty items. The *L*_1_ penalty term was originally proposed by the literature [28] when the *L*_1_ penalty term was combined with the ridge regression term in the optimization problem transformed by principal component analysis. It can constitute an elastic net penalty item. The penalty characteristic of the elastic net penalty item is between the *L*_1_ penalty item and the *L*_2_ penalty item. The *TL*_1_ penalty term is based on the literature [29] with a certain transformation in the form of the *L*_1_ penalty term, and new parameters are added to the numerator and denominator to control its characteristics between the *L*_0_ penalty term and the *L*_1_ penalty term. However, the essence of sparse principal component analysis is still aimed at processing linear data, and the processing of nonlinear data has not yet achieved the desired effect.

In view of the situation that nonlinear data cannot be handled well, kernel principal component analysis can solve this problem well. For high-dimensional feature space, this method uses the kernel function to project sample points from the original space with a linear distribution. This method and PCA have been combined naturally. This obtains sample points that are linearly distributed in high-dimensional space and finally uses principal component analysis in this space. Through the addition of the kernel function, the principal component analysis problem of nonlinear data is successfully solved. At the same time, it avoids the difficulty of not being able to obtain an explicit projection function by constructing a kernel matrix. This successfully transformed the way of analyzing the data covariance matrix into the way of analyzing the kernel matrix.

In this section, this article will focus on giving another extension method based on principal component analysis. This method is also an improved method based on the thinking of other expansion methods of principal component analysis in this article. The sparse kernel principal component analysis based on the *TL*_1_ penalty term is based on the thinking of sparse penalty and kernel principal component analysis. After learning sparse principal component analysis and kernel principal component analysis, it is logical to think of merging the two to get a sparse kernel principal component analysis in a high-dimensional space. In this way, the linear transformation of nonlinear data can be realized, and the sparse principal components can also be obtained in the feature space. This highlights the important variables and weakens the influence of some variables on the overall result, which has a simple and robust effect. Therefore, based on the method research on the principal component analysis, this paper proposes a sparse kernel principal component analysis.

#### 3.1.2. Improvement Strategy

The sparse kernel principal component analysis with *TL*_1_ penalty term combines the characteristics of the kernel principal component analysis as well as the sparse penalty, which can be implemented on nonlinearly distributed data. Its idea is similar to the sparse principal component analysis. What is considered in the sparse principal component analysis is to compare and sparse the linearly distributed data subjected to principal component analysis in the original space. When the covariance matrix is used to decompose to obtain the load, it is converted into an optimization problem, and then a penalty term is added to approach the partial load to zero. Therefore, through similar considerations, the original variables are transformed in the nuclear principal component analysis. At the same time, the eigenvalue decomposition of the sample point covariance matrix in the high-dimensional eigenspace is required. However, since the projection function cannot be obtained explicitly, it is finally transformed into the decomposition of the kernel matrix. Therefore, when the kernel matrix is eigen-decomposed, it can be transformed into an optimization problem. Then, we add the *TL*_1_ penalty term to this optimization problem, so that the new coordinates of the sample points in the feature space are sparse.

The characteristic equation for solving the kernel principal component analysis is

(1) $$\begin{array}{c} Kv_{i}=f_{i}v_{i}, \end{array}$$

where *K* is the kernel matrix and *f*_*i*_ is the eigenvalue corresponding to the eigenvector *v*_*i*_.

Then, the above equation can be written as an optimized regression model:

(2) $$\begin{array}{c} {\hat{v}}_{i}=\underset{v_{i}}{\text{arg}\min}K-f_{i}v_{i}v_{i2}^{T2}. \end{array}$$

Assuming *ν*_*i*_=*f*_*i*_*v*_*i*_ and *α*_*i*_=*v*_*i*_, then equation (2) can be written as

(3) $$\begin{array}{c} \left({\hat{\alpha }}_{i},{\hat{\nu }}_{i}\right)=\arg\min K-\nu_{i}\alpha_{i2}^{T2}. \end{array}$$

So far, this paper has transformed the principal component loading problem in nuclear principal component analysis into a regression optimization model. Therefore, the *TL*_1_ penalty term can be added to the above optimization problem, so that *ν*_*i*_ can be sparse. The model based on *TL*_1_ penalty term sparse kernel principal component analysis is obtained as

(4) $$\begin{array}{c} {\hat{\nu }}_{i}=\underset{\nu_{i}}{\text{arg}\min}K-\nu_{i}\alpha_{i2}^{T2}+\lambda_{1}\frac{\left(b+1\right)\nu_{i1}}{b+\nu_{i1}}, \end{array}$$

where *λ*_1_ and *b* are the parameters that need to be customized.

So far, this paper has given a sparse kernel principal component analysis model based on the *TL*_1_ penalty term. It can be seen from this model that its idea is similar to sparse principal component analysis. The biggest difference is that sparse principal component analysis is aimed at the covariance matrix of the data. The sparse kernel principal component analysis based on the *TL*_1_ penalty term is aimed at the mapped kernel matrix. From the perspective of the threshold iteration method, the threshold iteration algorithm for solving the sparse kernel principal component analysis based on the *TL*_1_ penalty term is as follows:

  Step 1: normalize the input matrix to get the output matrix *X*. Step 2: use the kernel method to process the matrix *X* to obtain the kernel matrix *K* in the high-dimensional space. Step 3: use general principal component analysis on the matrix *K* to get the load matrix *V*. Step 4: let *A*′ be the first *k*-dimensional column vector of the load matrix *V*; that is, *A*′=*V*[, 1 : *k*]. Step 5: let the initial coefficient of the *i*-th principal component be *x*_*i*_, given reasonable parameter values. Step 6: compare the size relationship between the parameters, and then perform the corresponding processing. Step 7: repeat Step 4 and Step 5; stop iterating when the conditions are met.

Therefore, this paper presents the basic model and algorithm of sparse kernel principal component analysis with *TL*_1_ penalty terms. It can be seen from the above steps that the penalty functions used are all *TL*_1_ penalty items. The process is basically similar to the solution of *TL*_1_ penalty term sparse principal component analysis. However, the most essential difference between the sparse kernel principal component analysis with *TL*_1_ penalty term and the sparse principal component analysis with the *TL*_1_ penalty term in the solution process is whether the original data has undergone a nonlinear transformation, that is to say, whether the data is mapped to the high-dimensional space. If it has undergone nonlinear processing, it is necessary to decompose and iterate the kernel matrix. Without this step, you only need to iteratively decompose the covariance matrix.

In general, an iterative algorithm is a more direct algorithm for solving optimization models. Iterative algorithms do not need to give a specific algebraic solution. Instead, it reaches a local optimal or global optimal solution by going through multiple cycles and iterations in a certain direction until it converges. However, there are many aspects that need attention in iterative algorithms. First, the selection of parameters in the iterative algorithm plays an important role and may even affect the selection of the final optimal solution. Therefore, a suitable method is needed to help the selection of parameters. Secondly, because the iterative algorithm achieves the purpose of solving by means of continuous loop iterations, so it is deeply affected by the number of iterations and accuracy. There may be a relatively large amount of calculation, especially when iterating all the elements in each vector of a matrix. Therefore, it can determine the appropriate threshold and number of iterations.

### 3.2. Improved BP Network

#### 3.2.1. Improved Particle Swarm Optimization

After using the proposed principal component analysis method to reduce the dimensionality of the features, this paper uses the BP network to model these features to predict the popularity of news public opinion.

Prediction accuracy is low due to random selection of initial threshold and weight parameters and the BP network's tendency to slip into the local minimum state. This results in poor performance. BP neural network model predictions can be enhanced by using an upgraded particle swarm technique to optimize the starting parameters. Model predictions can be made using a more accurate BP model that has been optimized for use in this study.

Particle swarm optimization (PSO) is an algorithm based on swarms that is extremely clever. The algorithm is easy to develop, is precise, and quickly converges to the best solution. It can effectively optimize various functions and is suitable for application in multiobjective constrained optimization problems. And it has better efficiency and practicability in solving various problems. At present, it has achieved good application results in constrained optimization, function optimization, various engineering design problems, and other fields.

Based on population dynamics, particle swarm optimization (PSO) is a population-based approach for solving optimization problems. One of its most fundamental aspects involves the creation of an initial group of particles in space using a randomized search process. Iteration is used to find the best solution for these particles, which begins with a random solution. There are three ways to express the attributes of a particle in an optimization problem: position, speed, and the fitness value. The fitness function can be used to calculate the particle's fitness value, which can be used to determine the particle's advantages and disadvantages. During each repetition, the particle updates itself by observing two points of departure. The individual extreme value is the one being sought by the particle, whereas the global extreme value is the one being sought by the entire group at the same time. Particles modify their speed and position by following these two extreme values, which each particle follows.

The basic principle of PSO mainly includes the initialization of particle swarm, the calculation for particle fitness, the update of particle speed and position, and the determination of termination conditions. The specific steps are as follows:

  Step 1: initialize the particle swarm. Step 2: calculate the objective function value, namely, fitness. Step 3: calculate fitness after the location update. The fitness is analyzed and compared with its individual extreme value Pbest. If fitness > Pbest, replace Pest with fitness. Step 4: compare fitness with the global extreme value Gbest. If fitness > Gbest, replace Gbest with fitness. Step 5: update the speed and position. Step 6: judgment of termination conditions. If the algorithm progresses until the end condition is met, the iterative calculation process is stopped, and the result is output at this time. Otherwise, the algorithm returns to Step 2.

A particle swarm algorithm can better solve combinatorial optimization problems. But this algorithm also has some shortcomings. Algorithm search accuracy is not very great and it is easy to get caught in the trap of local minimum solutions when using it. A modified particle swarm method using inertia weight has been developed to address these problems; in this way, the algorithm's search performance can be improved and it can avoid getting stuck in a local optimal scenario.

Among the various parameters of PSO, inertia weight *w* is an important parameter. This parameter affects the local optimal ability as well as the global optimal ability, and its value can reflect the speed of the particle's flight. If, in the algorithm parameter setting, the value for inertia weight *w* increases, the flying speed will increase, which can enhance the global search capability. On the contrary, if the value of *w* is reduced, the flying speed of the particles will slow down, which is beneficial to improve the local searchability. Therefore, it is necessary to select an accurate inertia weight *w*, so that the particles have balanced searchability, and effectively improve the exploration ability of the algorithm.

At present, many scholars have proposed improved methods for inertial weights, such as random weighting algorithms and fuzzy inertial weighting algorithms. One of the more common algorithms uses the method of linearly decreasing weights. Aiming at the characteristic that the PSO algorithm is easy to mature prematurely, literature [30] proposed a linear decreasing weight method. By introducing a linear transformation weight, the inertia weight decreases linearly from large to small. The formula for change with the number of iterations is as follows:

(5) $$\begin{array}{c} w=\frac{w_{\max}-\left(w_{\max}-w_{\min}\right)t}{t_{\max}}, \end{array}$$

where *w*_max_ is the maximum inertia weight, *w*_min_ is the minimum inertia weight, *t* represents the current iteration, and *t*_max_ is the maximum iteration.

Through analysis, although the introduction of linear inertia weight can make the PSO algorithm adjust the particle's optimization ability, this algorithm has some shortcomings. First of all, in the early stage of operation, due to the large value of the inertial weight *w* parameter, it is conducive to the global search of particles. If the optimal point is detected in the early stage of the algorithm, it is hoped that the algorithm will quickly converge to its optimal point. However, the linear decreasing characteristic of the inertia weight *w* slows down the convergence speed of the algorithm. Secondly, in the late running of the algorithm, as *w* decreases, the global search ability gradually decreases, which is easy to fall into the local optimal situation at this time.

Aiming at the shortcomings, an improved method of nonlinear decrease of inertia weight is proposed on the basis of the algorithm to improve the shortcomings. This algorithm is described as

(6) $$\begin{array}{c} w=w_{\max}-\left(w_{\max}-w_{\min}\right)\times \;\tan\left(\frac{\pi t}{4t_{\max}}\right). \end{array}$$

In the iterative process of the algorithm, when *t* is small, the global search capability can be guaranteed. Then, with the increase of *t*, local search ability can be guaranteed, so the algorithm has strong balanced searchability. Through the above-mentioned improved method, the nonlinear dynamic inertia weights better realize the balance between the global search and the local search. This improves the performance in convergence speed and global optimization.

#### 3.2.2. Improvement Strategy for BP

**Figure 1:**
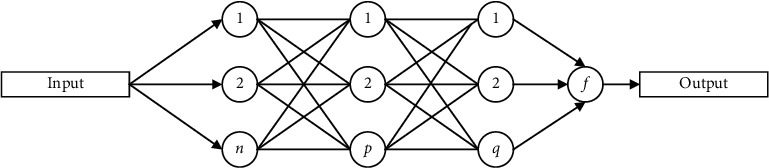
Structure of the BP network.

BP network is an algorithm based on gradient descent, as shown in Figure 1. As a result, the training effect of the network model will be negatively impacted by its inability to search globally. This cannot provide a more effective training method. Global optimization and simultaneous search are two features of the particle swarm algorithm. This has the potential to enhance global convergence. Neural networks can be improved by using this technique in the neural network algorithm. The particle swarm algorithm's global optimization ability is merged with the neural network's advantages by comparing their properties. It is possible to improve the learning and training speed of the network by establishing a BP neural network model using the particle swarm algorithm and optimizing the initial weights and threshold parameters of the BP network.

PSO may be used to optimize the initial parameters of a BP neural network model by integrating the particle swarm method into the model. Iterative particle optimization can be used to find the best parameters for the BP network. When it comes to the PSO algorithm, the BP neural network weights and thresholds are the flying particles of dimension two. In order to find the most optimal particles, you must first select the best initial parameters for the BP network. Additionally, iterative particles constantly adjust their velocity, position, and initial characteristics in space. Searching for the ideal particle is done in accordance with the function's optimization aim so that the particle's optimal parameter can be determined. The fitness value of a particle is the difference between the projected output of the BP model and the expected output, which is the optimization objective function. The fitness function is

(7) $$\begin{array}{c} f=\frac{1}{n}\sum_{i=1}^{n}\left|y_{i}-p_{i}\right|, \end{array}$$

where *n* is the number of samples, *y*_*i*_ is the actual output, and *p*_*i*_ is the predicted output.

Through the continuous update for the position, velocity, weight, and threshold, we determine the parameter value when the particle obtains the optimal fitness, assign the parameter value, and use the parameter optimized model for learning and training.

The principle of using the PSO algorithm to optimize the BP network algorithm mainly includes three parts: determining BP network structure, the PSO algorithm optimizing BP network parameters, and BP network training and prediction. The specific steps are as follows. (1) First, initialize the parameters for the model, including the number of network layers and learning rate of BP network, the number of populations of particle swarm algorithm, inertia weight, and the number of iterations. (2) Calculate and compare the fitness value. The output error of BP network model training is used as the fitness objective function to obtain the fitness value. (3) According to the fitness function, the improved particle swarm algorithm is used to iteratively search the space and update speed and position. (4) Determination of the termination condition of particle population update: when the algorithm reaches the maximum iterations, the loop iterative calculation process is stopped, the optimal fitness value is determined, and the optimal initial parameters are obtained. (5) Assign the optimized parameters to the BP network as initial weight and threshold parameters of the BP model. Combined with the sample data of news and public opinion, the optimized BP model is used to train until the entire model meets the convergence accuracy. (6) Use the trained BP model to predict the popularity of news public opinion.

## 4. Experiment and Discussion

### 4.1. Dataset

This article self-made two data sets DS1 and DS2. DS1 comes from online news published by Mashable Publishing House, and a total of 1389 news data are selected. DS2 comes from news published by Facebook, and a total of 1473 news data are selected. The specific distribution of the training set and test set is shown in Table 1. For each piece of news data, a 60-dimensional feature is extracted, and the dimensionality is reduced to a 20-dimensional feature vector through an improved principal component analysis algorithm. Each piece of news is divided into two categories: popular and unpopular. In this work, precision, recall, and *F*1 score are utilized to evaluate the performance of prediction.

### 4.2. Evaluation of Model Convergence

**Figure 2:**
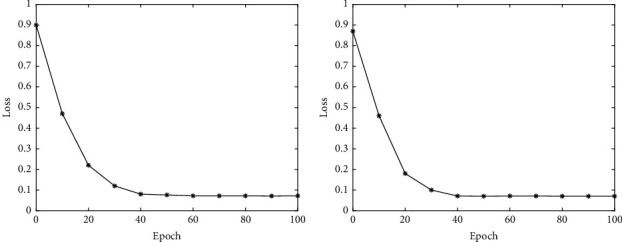
The training loss on DS1 and DS2.

In the BP network, whether the model converges is an important indicator for evaluating network performance. If the model fails to converge, subsequent predictions are meaningless. Therefore, this article first compares the training loss of the proposed network on two data sets. Experimental results are illustrated in Figure 2.

**Figure 3:**
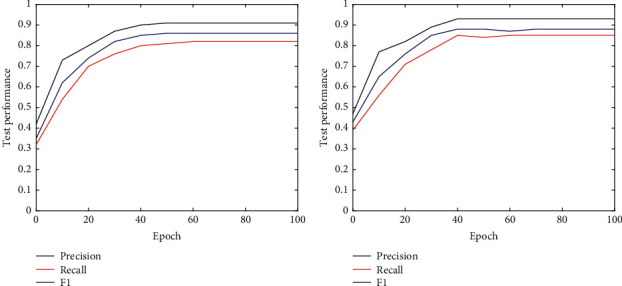
The test performance on DS1 and DS2.

As the training progresses, the loss of the network gradually decreases. And at the 40th epoch, the loss is basically not decreasing, indicating that the network has reached a state of convergence. Besides the training loss, this work also compares the test performance on two datasets. Experimental results are illustrated in Figure 3.

Similarly, when the training iteration is 40 epochs, the performance of the network on the test set tends to converge, which can obtain 0.91 precision, 0.82 recall, and 0.86 *F*1 on DS1, 0.93 precision, 0.85 recall, and 0.88 *F*1 on DS2. In summary, the designed network can finally converge and make stable and efficient predictions.

### 4.3. Evaluation on Improved PCA

**Figure 4:**
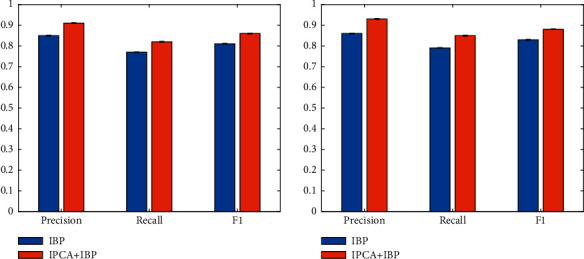
Evaluation of improved PCA.

In this work, an improved PCA is proposed to reduce the dimensionality of features. To prove that this method can effectively improve the performance, the performance when using the improved PCA (IPCA) is compared with that of the improved BP (IBP) network alone. The experimental results are illustrated in Figure 4.

Combining the IPCA method with the BP network can effectively improve the performance. On the DS1 dataset, compared with not using the IPCA method, the performance improvement obtained is 6%, 5%, and 5% on three indexes. On the DS2 dataset, compared with not using the IPCA method, the performance improvement obtained is 7%, 6%, and 5% on three indexes. To further illustrate the superiority of the IPCA method, it is compared with the traditional PCA. Experimental results are illustrated in Table 2.

Obviously, compared with the PCA algorithm, the IPCA method can further improve the performance of the BP network. This proves the reliability and effectiveness of the IPCA method.

### 4.4. Evaluation of Improved PSO

**Figure 5:**
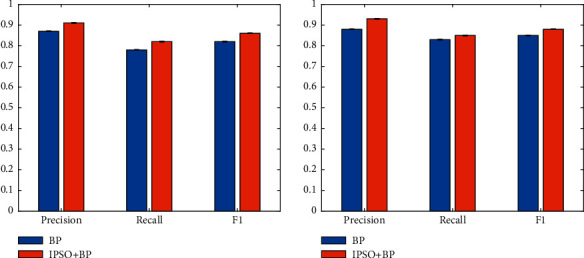
Evaluation of improved PSO.

In this paper, an improved PSO is proposed to optimize the BP network. To prove that this method can effectively improve the performance of the BP network, the performance when using the improved PSO (IPSO) is compared with that of the BP network alone. The experimental results are illustrated in Figure 5.

It can be seen that combining the IPSO method with the BP network can improve performance. On the DS1 dataset, compared with not using the BP method, the performance improvement obtained is 4%, 4%, and 4% on three indexes. On the DS2 dataset, compared with not using the IPSO method, the performance improvement obtained is 5%, 2%, and 3% on three indexes. To further illustrate the superiority of the IPSO method, it is compared with the traditional PSO method. Experimental results are illustrated in Table 3.

Obviously, compared with PSO, the IPSO method can further improve the performance of the BP network. This proves the reliability and effectiveness of the IPSO method in this paper.

### 4.5. Comparison to Other Methods

To illustrate the effectiveness of our strategy, this article compares our method with other methods. Experimental results are illustrated in Table 4.

Obviously, compared with other methods, our method can obtain the best performance improvement. This demonstrates the correctness of the method in this article.

## 5. Conclusion

With the development of the Internet, news and public opinion have exploded. It is a very important subject to extract and study the characteristic attributes that determine the popularity of news public opinion and establish a network model to predict the popularity of news public opinion. The main work of this paper has the following two contents. The first is to realize the sampling of news and the extraction of attribute features. Then, considering the nonlinear relationship between features, an improved principal component analysis method is proposed to analyze the correlation of features. In this way, important features can be selected from many irrelevant features, effectively reducing the original high-dimensional features. Second, neural networks are examined for their ability to anticipate public opinion on news stories. By using BP, we can mine data more effectively. The BP neural network, on the other hand, has a number of limitations and uses an improved approach for optimizing the initial parameters of the BP neural network, which successfully compensates for the BP neural network's flaws. Finally, a prediction model for the popularity of news public opinion is built using the BP network with optimal parameters. The new neural network model described in this paper can accurately predict the popularity of news public opinion, as demonstrated by experimental findings.

## Figures and Tables

**Table 1:** The division of training set and test set.

Dataset	Training set	Test set
DS1	1041	348
DS2	1104	369

**Table 2:** Comparison of IPCA and PCA.

Method	DS1			DS2		
	Precision	Recall	*F*1	Precision	Recall	*F*1
PCA + IBP	0.89	0.80	0.83	0.90	0.82	0.84
PCA + IBP	0.91	0.82	0.86	0.93	0.85	0.88

**Table 3:** Comparison of IPCA and PCA.

Method	DS1			DS2		
	Precision	Recall	*F*1	Precision	Recall	*F*1
PSO + BP	0.88	0.80	0.84	0.91	0.84	0.85
IPSO + BP	0.91	0.82	0.86	0.93	0.85	0.88

**Table 4:** Comparison to other methods.

Method	DS1			DS2		
	Precision	Recall	*F*1	Precision	Recall	*F*1
DT	0.75	0.67	0.71	0.79	0.69	0.73
SVM	0.81	0.72	0.75	0.81	0.78	0.75
BP	0.85	0.77	0.79	0.86	0.83	0.82
Ours	0.91	0.82	0.86	0.93	0.85	0.88

## Data Availability

The datasets used are available from the corresponding author on reasonable request.
